# Sebaceous carcinoma of the trunk and extremities: Epidemiology and treatment patterns in the United States

**DOI:** 10.1016/j.jdin.2024.03.012

**Published:** 2024-03-28

**Authors:** Miles Grunvald, Rachel Chang, Samantha Terranella, Ethan Ritz, Parul Kathuria Goyal, Cristina O’Donoghue

**Affiliations:** a Department of Surgery, Rush University Medical Center, Chicago, Illinois; b Department of Dermatology, Rush University Medical Center, Chicago, Illinois

**Keywords:** epidemiology, lymph node, medical dermatology, National Cancer Database, oncology, sebaceous carcinoma, surgery, treatment

## Abstract

**Background:**

Sebaceous carcinoma is a rare cancer, and little is known about its current epidemiology and treatment. This is particularly true for sebaceous carcinomas of the trunk and extremities.

**Objective:**

We present a database analysis of sebaceous carcinoma cases to further delineate demographics, location, tumor characteristics, and treatment modalities among patients diagnosed with these tumors.

**Methods:**

The National Cancer Database was queried for cases of sebaceous carcinoma between 2004 and 2016. 3211 cases were analyzed for descriptive and comparative statistics.

**Results:**

Twenty-six percent of sebaceous carcinomas were found on the trunk and extremities. Tumors on the trunk and extremities were more likely to be larger than tumors on the head and neck, with 8% being greater than 50 mm (*P* < .001). Tumors on the trunk and extremities were more likely to be well differentiated (*P* < .001) and have fewer lymph node metastases (*P* < .001). Surgery was the primary treatment modality for tumors, followed by radiotherapy and rarely chemotherapy.

**Conclusions:**

Sebaceous cancer is a poorly understood entity. We demonstrated that trunk and extremity tumors tend to be larger and more differentiated than those of the head and neck. Treatment practices are varied at this time, but surgery is the primary modality.

**Capsule Summary**

• Information on epidemiology and treatment of sebaceous carcinoma is limited, especially for those originating from the trunk and extremities.
• Sebaceous carcinoma of the trunk and extremities were larger and were more likely to be well-differentiated and have fewer lymph node metastases. Surgery is the primary treatment modality, followed by radiotherapy.

## Introduction

Sebaceous carcinoma is a rare oncologic process arising from dermal appendages.^1^^,^^2^ It is uncommon with an incidence thought to be less than 1/1000,000 person-years and has a predominance in older, white male patients.^3^, ^4^, ^5^ Sebaceous carcinoma has a proclivity for the eyelid, where it has traditionally been associated with a more aggressive course.^4^ It is frequently associated with genetic diseases related to DNA mismatch repair proteins, most commonly Muir-Torre syndrome.^3^

There are no current National Comprehensive Cancer Network guidelines for the management of sebaceous carcinoma, although there is a recently published guideline from the Committee on Invasive Skin Tumor Evidenced Based Recommendations. The current standard of care for this disease is primarily a surgical approach aimed at complete resection of cancerous tissue. Wide local excision is typically performed with 1-centimeter margins, but Mohs surgery has also been utilized for this cancer as well, particularly in cosmetically sensitive areas.^6^ The role for radiotherapy in these patients has not been clearly defined. There are no specific systemic therapies recommended for metastatic disease. Decisions regarding systemic therapy should be discussed with a multidisciplinary team and newer targeted therapies, such as anti-programmed death-1 inhibitors, should be considered.^7^, ^8^, ^9^

The necessity of lymph node sampling and management in patients diagnosed with sebaceous carcinoma is not clear. The Committee on Invasive Skin Tumor Evidenced Based Recommendations guidelines do not recommend routine sentinel lymph node biopsy but concede that there may be some factors that predispose to lymphatic spread. Recent data suggest that periocular sebaceous carcinoma may metastasize to the lymph nodes in up to 4% of cases and that lymph node sampling would potentially be advantageous in this population.^10^^,^^11^ Much of the research into this aspect of sebaceous carcinoma has utilized the Surveillance, Epidemiology, and End Results Program database.^4^^,^^12^, ^13^, ^14^ Furthermore, current research has focused predominantly on ocular, head, and neck sebaceous carcinoma with less of an emphasis on truncal and extremity disease.

Our study utilizes the National Cancer Database (NCDB) to identify current trends in the diagnosis of sebaceous carcinoma and lymph node management and to more specifically investigate patterns among cases diagnosed on the trunk and extremities.

## Methods

Data were extracted from the NCDB between 2004 and 2016 for this study. The NCDB is a nationwide database in the United States that collects information on oncologic processes and outcomes. It captures approximately 70% of all invasive cancers from more than 1500 participating hospitals. The NCDB is a joint project between the Committee on Cancer and American College of Surgeons.^15^ All data from the NCDB are de-. Following formal IRB exemption, a participant user file from the NCDB was obtained. Patients with a primary diagnosis of sebaceous carcinoma greater than 18 years old were included in this study.

Descriptive statistics were used to describe patient demographics and clinical characteristics. Categorical data were reported by numbers and percentages, and group differences were measured by Pearson χ^2^ tests or Fischer exact tests. Analytic statistical tools were conducted to identify tumor specific patterns and treatments patterns. Cases were grouped by sex, racial group, insurance status, and anatomic tumor sites. Cases were further stratified by tumor size and grade, nodal stage, and treatment regimen according to tumor location. Data were analyzed using analysis of variance; a 2-tailed *P* value of less than .05 was considered statistically significant. At times, data from the NCDB was incomplete; namely, pathologic nodal stage was not always reported. Analyses were completed with available data, and we have indicated where data was lacking in the results. All statistical analysis was performed with STATA software (version 13.0, StataCorp LP).

## Results

We report a total of 3211 sebaceous carcinoma cases identified within the NCDB registry from 2004 to 2016. Demographic information is reported in Table I. Of the 3211 patients, 59% were male and 40% were female. The average age was 69 years old. Nearly 90% of patients reported their race as white. Primary tumor location was most frequently on the eyelid (31%); however, 26% were located on the trunk, upper extremity, or lower extremity (Table II).

**Table I:** Demographics

	No. of patients (%)
Total	3211
Sex	
Male	1895 (59.0)
Female	1316 (41.0)
Race	
White	2887 (89.9)
Black	110 (3.4)
American Indian, Aleutian, or Eskimo	15 (0.5)
Asian	87 (2.71)
Other/unknown	112 (3.49)
Hispanic	
Yes	154 (4.8)
No	2886 (89.9)
Unknown	171 (5.3)
Insurance status	
Not insured	67 (2.1)
Private	1023 (31.9)
Medicaid/Medicare/other governmental	2041 (63.6)
Unknown	80 (2.5)
Primary site	
Eyelid	989 (30.8)
Face/ear/scalp/neck/lip	1365 (42.5)
Trunk	606 (18.9)
Extremities	218 (6.8)

**Table II:** Tumor size and grade by primary site location

	Trunk and extremities (%)	Face/ear/scalp/neck/lip (%)	Eyelid (%)	Total
Size				
Unknown	281 (34.1)	522 (38.2)	535 (54.1)	1338
0-9 mm	127 (15.4)	409 (30.0)	212 (21.4)	748
10-29 mm	247 (30.0)	334 (24.5)	188 (19.0)	769
30-50 mm	101 (12.3)	69 (5.1)	35 (3.5)	205
>50 mm	68 (8.3)	31 (2.3)	19 (1.9)	118
Grade				
Well differentiated	171 (20.8)	230 (16.8)	51 (5.2)	452
Moderately differentiated	74 (9.0)	102 (7.5)	101 (10.2)	277
Poorly differentiated	63 (7.6)	153 (11.2)	208 (21.0)	424
Undifferentiated	7 (0.8)	16 (1.2)	18 (1.8)	41
Not determined/not stated	509 (61.8)	864 (63.3)	611 (61.8)	1984

Tumor size was recorded in over 50% of cases. Tumors found on the trunk and extremity tended to be larger in size than those found on the head and neck, with 8% being greater than 50 mm (analysis of variance , *P* < .001). Despite this size difference, trunk and extremity sebaceous carcinomas were not found to be less differentiated when compared to those of the head and neck, albeit the majority of tumors did not have their pathology recorded (Table II) (analysis of variance , *P* < .001).

Lymph node involvement was uncommon, occurring in 6% of cases with recorded primary sites. Stratification was conducted by primary site, and primary tumor location at the eyelids had a trend toward increased nodal involvement (Table III). Of cases where pathologic node status was recorded, trunk and extremity tumors demonstrated less nodal involvement when compared to those of the eyelid (*X*^2^ = 12.8, *P* < .001).

**Table III:** Nodal stage by primary tumor location

	Stage			
	N0 (%)	N1 (%)	N2 (%)	Total
Eyelid	222 (91.4)	21 (8.6)	0 (0)	243
Face/ear/scalp/neck/lip	361 (93.3)	20 (5.2)	6 (1.6)	387
Trunk/extremities	268 (97.5)	5 (1.8)	2 (0.7)	275
Total	851 (94.0)	46 (5.1)	8 (0.9)	905

There is an inverse relationship between tumor differentiation and nodal metastasis. Of 105 patients with well-differentiated tumors and recorded nodal status, none had nodal metastasis. This is in juxtaposition to patients with poorly and undifferentiated tumors with recorded nodal status, who were found to have a 5% nodal involvement.

Management of lymph nodes with biopsy or dissection was frequent in this cohort; 13% of patients were recorded as having received a lymph node biopsy and 2.6% were recorded as having undergone a lymph node dissection (as defined as having greater than 9 lymph nodes harvested).

There was a wide variety in primary site management of sebaceous carcinoma. The vast majority (86%) received treatment with surgery alone. These procedures included “local excision,” “biopsy followed by gross excision,” “wide excision,” and “Mohs” amongst others. Over 6% received radiotherapy as part of their treatment regimen. There was a minimal use of systemic chemotherapy in our cohort (Table IV).

**Table IV:** Treatment regimen by tumor location

Treatment combination	Trunk and extremities (%)	Face/ear/scalp/neck/lip (%)	Eyelid (%)	Total (%)
Surgery alone	740 (89.8)	1160 (85.0)	853 (86.2)	2753 (86.4)
Surgery and radiation	32 (3.9)	87 (6.4)	43 (4.3)	162 (5.08)
Surgery, radiation, and chemotherapy	2 (0.2)	7 (0.5)	8 (0.8)	17 (0.5)
Radiation alone	1 (0.1)	13 (1.0)	9 (0.9)	23 (0.8)
Chemotherapy and radiation	1 (0.1)	4 (0.3)	2 (0.2)	7 (0.3)
Surgery and chemotherapy	8 (1.0)	2 (0.1)	15 (1.5)	25 (0.8)
Chemotherapy alone	0 (0.0)	4 (0.3)	1 (0.1)	5 (0.2)
None	19 (2.3)	45 (3.3)	27 (2.7)	91 (3.0)
At least 1 unknown	21 (2.5)	43 (3.2)	31 (3.1)	95 (3.0)

## Discussion

Sebaceous carcinoma is a rare and poorly understood cancer derived from the dermal appendages. In this study, we present the largest retrospective cohort of cases to date. Further, this is the first study to assess trends in sebaceous carcinoma epidemiology and treatment using the NCDB.

Our study has demonstrated and confirmed some findings that were previously identified using the Surveillance, Epidemiology, and End Results Program database. Previous authors have reported under registration of patients in Surveillance, Epidemiology, and End Results Program registries as a source of limitation.^14^ Supplementing data with other databases such as the NCDB that may include a broader coverage of cancer cases will build on existing literature. Patients who are older, white, and male have consistently demonstrated a higher incidence of sebaceous carcinoma.^14^^,^^16^ Importantly, we confirm that sebaceous carcinoma of the eyelid does seem to have a slightly higher risk of nodal metastasis.^17^^,^^18^ We have shown that there is overall a low nodal metastasis rate in sebaceous carcinoma and that well-differentiated adenocarcinoma carries a low risk of nodal involvement.

Sebaceous carcinoma of the trunk and extremities has received less focus in the literature than tumors of the head and neck. In an epidemiological study of sebaceous carcinoma using cancer registries from various countries, Wu et al report that ocular sebaceous carcinoma has been the primary focus in reporting, documentation, and analysis, with registries in Asia focusing on ocular sebaceous carcinoma only.^19^ There remains a gap in literature in understanding tumor characteristics of sebaceous carcinoma of the trunk and extremities. This study shows that tumors of the trunk and extremity are generally larger than those of the head and neck but do not seem to be less differentiated. Generally, these cancers seem to have less nodal involvement than those of the eyelid.

Nodes in this cohort were routinely assessed with biopsy. However with the data, we are not able to determine if these were radiographically guided node biopsies, biopsy of palpable nodes, or sentinel lymph node biopsy. A smaller group of patients (2.55% of this cohort) underwent lymph node dissection as defined by a lymph node harvest of >9 nodes, a cutoff previously chosen for dissection in prior breast literature utilizing the NCDB. This lymph node dissection rate was roughly consistent with the 1.74% lymph node involvement. Most cases reported “unknown” lymph node status.

We present the first characterization of current trends in the treatment of sebaceous carcinoma. Notably, a subset of patients in our cohort received radiotherapy. Current recommendations suggest the consideration of radiotherapy in patients with node-positive disease to treat lymph node basins in conjunction with primary tumor resection, or for palliation of primary site in patients who cannot undergo surgery. Interestingly, we observed instances of nonguideline driven radiotherapy, primarily among patients with N0 disease who initially underwent surgery. However, the reason for radiotherapy inclusion amongst these patients could not be interpolated from the data set. Future research can help elucidate factors influencing decision to administer radiotherapy in patients with N0 disease following initial surgery. Additionally, we identified guideline-consistent use in patients with N1 disease who underwent primary resection.

Future research into treatment of sebaceous carcinoma will be largely dependent on detailed reporting of disease stage, severity, treatment regimens and outcomes. Clinical trials are exceedingly challenging in the study of this oncologic entity due to its rare incidence and variety of presentation and location. Retrospective tools and databases should be honed to help identify best practices in sebaceous carcinoma care.

This project, like previous retrospective database studies assessing sebaceous carcinoma, is limited by incomplete data. Increased fidelity and completeness of data collection will be integral to the integrity future study. The NCDB database may include selection bias from reporting facilities and lacks important outcomes data such as recurrence, disease free survival, and complications associated with treatment regimens. Limitations also included those inherent to a retrospective study design.

## Conclusion

Sebaceous carcinoma is a rare and aggressive cancer with much work to be done regarding the diagnosis and treatment. To our knowledge, this is the largest retrospective cohort of sebaceous carcinoma cases to date, and the first study to assess trends in sebaceous carcinoma epidemiology and treatment using the NCDB database. We have found that cancer of the trunk and extremities were larger than those of the head and neck and were more likely to be well differentiated. Nodal metastasis was rare but associated with poor differentiation and primary tumor location on the eyelid. Current management is predominantly with surgical excision, consistent with current guidelines, but there is regular use of radiotherapy among a subset of patients as well.

## Conflicts of interest

None disclosed.
